# Rural–urban differences in osteoporosis and sarcopenia prevalence among Gambian older adults: a pilot study

**DOI:** 10.1093/jbmr/zjaf130

**Published:** 2025-09-22

**Authors:** Mícheál Ó Breasail, Ayse Zengin, Camille Pearse, Isatou Drammeh, Ramatoulie Janha, Landing Jarjou, Peter R Ebeling, Ann Prentice, Kate A Ward

**Affiliations:** Department of Medicine, School of Clinical Sciences, Faculty of Medicine, Nursing and Health Sciences, Monash Medical Centre, Monash University, Clayton, Victoria, 3168, Australia; Department of Medicine, School of Clinical Sciences, Faculty of Medicine, Nursing and Health Sciences, Monash Medical Centre, Monash University, Clayton, Victoria, 3168, Australia; MRC Lifecourse Epidemiology Centre, University of Southampton, Southampton General Hospital, Tremona Road, Southampton, SO16 6YD, United Kingdom; MRC Unit, The Gambia at London School of Hygiene and Tropical Medicine, Banjul, The Gambia; MRC Unit, The Gambia at London School of Hygiene and Tropical Medicine, Banjul, The Gambia; MRC Unit, The Gambia at London School of Hygiene and Tropical Medicine, Banjul, The Gambia; Department of Medicine, School of Clinical Sciences, Faculty of Medicine, Nursing and Health Sciences, Monash Medical Centre, Monash University, Clayton, Victoria, 3168, Australia; MRC Unit, The Gambia at London School of Hygiene and Tropical Medicine, Banjul, The Gambia; MRC Epidemiology Unit, Institute of Metabolic Science, University of Cambridge School of Clinical Medicine, Cambridge, CB2 0SL, United Kingdom; MRC Lifecourse Epidemiology Centre, University of Southampton, Southampton General Hospital, Tremona Road, Southampton, SO16 6YD, United Kingdom; MRC Unit, The Gambia at London School of Hygiene and Tropical Medicine, Banjul, The Gambia

**Keywords:** aging, analysis/quantitation of bone, DXA, bone QCT/μCT, general epidemiological studies

## Abstract

Rural–urban BMD differences are well described in high-income countries, typically with higher BMD in rural areas. However, despite rapid urbanization and longevity across Africa, such data remain scarce. This study compares bone and muscle health in older adults living in rural and urban Gambia. Participants aged ≥55 yr from rural (*n* = 209) and urban (*n* = 101) communities were measured with DXA (TH, FN, and LS) and peripheral QCT (pQCT; diaphyseal and epiphyseal radius and tibia). Outcomes were DXA areal BMD (aBMD), BMC, bone area (BA); pQCT total volumetric BMD (vBMD), trabecular vBMD, bone strength indices (BSIc), cross-sectional area (CSA), BMC, and cortical vBMD. Osteoporosis (NHANES III, T-score < −2.5) and sarcopenia (revised European Working Group on Sarcopenia in Older People [EWGSOP2] appendicular lean mass [ALM] and handgrip strength [HGS]) prevalence were computed. Linear regression was used to describe rural–urban differences in DXA and pQCT outcomes (1) age-adjusted and (2) adjusted for age and fat mass index (FMI). Osteoporosis at either the FN or TH was more prevalent in urban men (20% vs rural 10%) and rural women (45% vs urban 31%). LS T-scores < −2.5 were more common in rural participants (men: 27% vs 14%; women: 61% vs 35%). Sarcopenia was also higher in rural participants (men: 30% vs. 18%; women: 18% vs 15%). Adjusted for age and FMI urban Gambians had lower BMC but greater BA at the FN and TH, while aBMD differed little. Urban men had lower adjusted tibial cortical vBMD but greater tibial diaphyseal and radial epiphyseal CSA. After adjustment, urban women had greater radial CSA and estimated strength. Our findings highlight that osteoporosis and sarcopenia are highly prevalent in older Gambian adults, with differences in rural–urban prevalence influenced by sex. Given ongoing nutrition transition and urbanization across Africa, larger population-based studies are urgently required to better inform targeted prevention strategies and interventions.

## Introduction

At present, the United Nations–defined region of sub-Saharan Africa has twice the number of adults aged ≥60 yr as northern Europe, and is projected to increase from approximately 46 million in 2015 to 157 million by 2050.^1^ Population aging is associated with the increasing burden of noncommunicable diseases, including those that impact the musculoskeletal system. This includes osteoporosis and sarcopenia, both contributing to significant morbidity and mortality in older people.^2^ While this epidemiological and economic burden is well described in high-income countries (HICs) across Europe, North America, and East Asia,^3^^,^^4^ data are lacking from low-income, resource-limited countries, particularly in Africa.^5^^,^^6^ Sparse and low-quality data make global comparisons of temporal trends and geographical differences in musculoskeletal health challenging.^7^ For example, global data on adult hip fractures suggest that the incidence in Africa and Asia is considerably lower than in age-matched White populations.^8–10^ However, recent data comparing osteoporosis prevalences in The Gambia, South Africa, and Zimbabwe with those in the United States and United Kingdom suggest that the burden of osteoporosis in Africa is more similar to that in HICs.^5^ Similarly, recent multiethnic hip fracture data from South Africa show incidence rates that are much higher than previously reported,^11^ with a predicted doubling of incidence over the coming decades. While ethnic differences in hip fracture incidence were similar to those elsewhere across the globe, the mortality rate associated with a hip fracture was much higher in Black individuals. Attempts to estimate the prevalence of sarcopenia in Africa have been hampered by limited data; the paucity of quality epidemiological, population-based studies; and hence a lack of applicable clinical definitions with (African) population-specific cutoffs.^12^^,^^13^

The Gambia, West Africa, is a low-income country undergoing economic transition coupled with rapid urbanization and nutrition transition. As of 2024, 64% of the population of The Gambia (total population, 2.42 million^14^) lives in urban areas, up from 48% in 2000.^15^^,^^16^ Over the same timeframe there has been a rapid increase in the number of overweight and obese people, in both urban and rural communities, although obesity remains more prevalent in urban areas.^17^^,^^18^ This has meant that The Gambia has had to contend with the simultaneous burdens of under- and overnutrition, in combination with micronutrient deficiencies, termed the “double or triple burdens” of malnutrition.^17–21^ Given that 50.6% of the population are of working age, this has important implications for economic development.^14^ Recently published nutrition data demonstrate food-group differences between rural and urban Gambian households.^21^ In contrast to many similar low- and middle-income countries (LMICs), there is a long history of research including musculoskeletal phenotyping across the life course in The Gambia.^22–24^ This research historically was limited to rural Gambia, although, in recent years, efforts have increased to focus on urban musculoskeletal health.^25^^,^^26^ This has presented the opportunity to explore putative rural-urban differences in musculoskeletal health, which may be driven by environmental exposures, occupational physical activity, and nutritional differences (including dietary patterns and dietary diversity). Previous investigations of Gambian rural–urban migration have suggested that bone health may differ according to rural vs urban dwelling.^27^ Therefore, it is plausible that, given these lifestyle disparities, the prevalence of osteoporosis and sarcopenia may differ.

A number of studies have explored rural–urban differences in BMD and other parameters related to bone strength; these were most recently synthesized in a 2015 systematic review and meta-analysis.^28^ Based on the available literature, the authors reported a different pattern or rural–urban differences within HICs and LMICs—that is, in HICs, BMD tended to be higher in rural areas, whereas for LMICs, there were fewer studies and the relationship was less clear.^28^ No data from Africa were included in this review. Given the more recent emergence of sarcopenia as a concept in gero-science it is perhaps unsurprising that there have been fewer studies focusing on sarcopenia and particularly on rural–urban comparisons of muscle health in LMICs.^12^^,^^29^ Therefore, the aims of this study in rural and urban Gambian women and men aged 55 yr and over were to compare the following: (1) the prevalence of osteoporosis and sarcopenia; (2) bone density, mass, geometry, and strength; (3) body composition and hand grip strength; and (4) to identify potential determinants of differences in the musculoskeletal phenotype of rural vs urban dwellers.

## Materials and methods

### Participants

The Gambian Bone and Muscle Aging Study (GamBAS-rural) is a prospective observational study in Black African men and women aged ≥40 yr (ISRCTN17900679). The study protocol for this cohort has been published.^13^ The target study sample for rural participants was 240 women and 240 men, identified using the Kiang West Demographic Surveillance System (KWDSS).^16^ Participants were recruited and stratified by sex and by 5-yr age bands to ensure equal distribution. The oldest age band was 75+ years. Rural participants had baseline measurements in 2011–2012 and were followed up 6–8 yr later (2017–2019). Rural data from this 2017–2019 follow-up were used in the present study. A group of urban participants, aged 50–80 yr, were recruited in 2019 using the same inclusion criteria as GamBAS and had a single assessment.^26^ Urban GamBAS participants were recruited by convenience sampling through community networks in Sukuta, West Coast Region, The Gambia. Ethnic group was self-reported. Assessments took place at the MRC Keneba (rural) and MRC Fajara (urban) bone imaging facilities. Ethics approval for all visits was granted by The Joint Gambia Government/MRC Unit The Gambia at London School of Hygiene and Tropical Medicine Ethics Committee (original reference, SCC1222; new reference, 28118). Only rural participants aged 55 yr and over were included in these analyses as this was the youngest age of urban residents. All women were postmenopausal.

#### Anthropometry

Height (cm) was measured to the nearest 1 mm using a wall-mounted stadiometer (Seca GmbH, Hamburg, Germany) and weight (kg) measured to the nearest 0.1 kg using a digital scale (Seca GmbH, Hamburg, Germany) while the participants wore light clothing without footwear. Waist and hip circumferences (cm) were obtained using a flexible tape. Subsequently, BMI (kg/m^2^) was calculated. Forearm and lower-leg length were measured to the nearest 1 mm using a tape measure: tibia length was measured from the distal edge of the medial malleolus to the tibial plateau; ulna length was recorded as the distance from the olecranon to the ulnar styloid process.

#### Physical function assessments

Grip strength was measured by hand dynamometer (Jamar Hand Dynamometer, IL, USA)^30^ with participants seated in an upright position with their arm supported on the armrest of the chair and wrist in a neutral position and the thumb facing upwards. Participants were instructed to exert maximal force. One practice effort was permitted followed by 3 test measurements. The outcome measured was force (kg) for the highest effort.

#### Bone and body composition imaging

A pair of DXA scanners (iDXA; GE-Lunar, Waltham, MA, USA) were used to perform whole body, dual femur, and LS scans. All image analysis used the manufacturer’s software (Encore, version 15). Total lean mass (LM), total fat mass (FM), in addition to subregional measures of LM used to derive appendicular LM (ALM); ALM index (ALMI = ALM/height^2^) and fat mass index (FMI = fat mass/height^2^) were assessed from whole body scans. Bilateral proximal femur scans were acquired for TH and FN areal BMD (aBMD), BMC, and bone area (BA) measurements. The femur scan with the lowest FN aBMD value was selected for inclusion, with TH values taken from the same side. Lumbar spine aBMD was taken as the mean value of L1 to L4; however, where degeneration or artifacts were present and all vertebrae could not be included, LS aBMD was recalculated (eg, L1 BMC + L2 BMC/ L1 BA + L2 BA) based on the remaining vertebrae providing 2 or more consecutive vertebrae were available. Neither an averaged LS BMC nor LS BA were derived in such cases and are not included in further analysis. Due to computer failure, LS data were not available for 51 rural participants in the present analysis, as reported previously.^26^ T-scores were calculated as per the International Society of Clinical Densitometry (ISCD) guidelines, using NHANES III data for T-score calculations.^17^

Two peripheral QCT (pQCT) scanners (XCT2000 and XCT2000L; Stratec Medizintechnik GmBH) were used. Scan acquisition parameters were as follows: voxel size of 0.5 × 0.5 mm, slice thickness of 2 mm, CT scan speed of 30 mm/s, and scout view scan speed of 40 mm/s. Measurements were obtained at 4% and 33%/38% of the limb length proximal to the distal endplate of the radius and tibia. All pQCT image analysis used the manufacturer’s software (version 6.2). At distal 4% sites, CALCBD analysis (contour mode 1, threshold 180 mg/cm^3^, peel mode 1) was used to calculate total cross-sectional area (CSA) and total and trabecular volumetric BMD (vBMD). Bone strength index of compression (BSIc) was subsequently calculated as total vBMD^2^ × total CSA. At proximal cortical-rich sites, CORTBD, separation mode 1, threshold 710 mg/cm^3^, was used to define cortical vBMD and area. Total CSA was defined at proximal sites at a threshold of 280 mg/cm^3^. Scans were qualitatively graded by visual inspection (M.Ó.B.) to assess their suitability for longitudinal analysis: scan slices with excessive movement or other artifacts were excluded (Table S1).

Quality assurance and quality control procedures for all scanners were as per manufacturer guidelines with phantoms scanned daily for quality assurance and weekly for quality control. These also monitor scanner drift and performance over time. Precision data at these facilities based on duplicate scans from 30 Gambian adults were as follows: DXA total hip precision was 0.7% and for pQCT was 0.3%-1.8% for bone measures at the tibia and 1.1%-6.4% at the radius. Both pairs of pQCT and iDXA scanners were cross-calibrated using in vivo data from 60 Gambian men and women.^31^

#### Osteoporosis and sarcopenia prevalence

Normal BMD, low bone mass, and osteoporosis were defined as per ISCD guidelines based on T-score calculated as above based on NHANES III.^17^ Low bone mass was defined as having a T-score between −1 and −2.5 and osteoporosis as a T-score below −2.5.

At present, there are no accepted sarcopenia definitions validated for use in West African, or any African, populations. Sarcopenia was defined as per the revised European Working Group on Sarcopenia in Older People (EWGSOP2) using sex-specific cutoffs for DXA ALM (males [M] <20 kg; females [F] <15 kg) and ALMI (M <7 kg/m^2^; F <6 kg/m^2^) in combination with grip strength (M <27 kg; F <16 kg).^32^ The decision to use EWGSOP2 rather than an alternative definition was based on previous work, which found ALM to be the best predictor of poor physical performance in rural participants.^13^

### Statistical analysis

All data analyses were conducted using R (version 4.3.1) and Rstudio (RStudio 2023.06.1 + 524). All analyses were conducted in men and women separately as per the original study design and due to the known between-sex bone and body composition differences. Data are summarized by cohort with quantitative variables expressed as mean and SD where normally distributed, or otherwise as median and IQR. Categorical variables are presented as frequencies with percentages. Comparisons of quantitative variables by cohort were performed using the Student’s *t* test where normally distributed or the Mann-Whitney *U* test if skewed. The chi-square test was used to compare categorical variables by cohort. For visualization and standardization across the varying bone and composition units, between-group differences in each variable are expressed as a sympercent ([difference/mean] × 100), derived by subtracting log_e_(urban) from log_e_(rural) and multiplying by 100.^33^

Sex-stratified linear regression models were used to explore rural–urban differences in DXA and pQCT bone outcomes after adjusting for age (model 1) and age and FMI (model 2). Due to a greater extent of missing data at the radius compared with the tibia, sensitivity analyses were performed to explore whether differences observed between limbs could be a result of different participants being included. Complete case analysis was performed (*n* = 212), in addition to the analysis of a subset who had data at either radial site (*n* = 293). DXA analyses were also repeated in the subset with LS aBMD (*n* = 255) to confirm if rural–urban patterns at the FN and TH were consistent. Further sensitivity for season defined as harvest (January–June) and hungry (July–December) impacted the observed relationships.

## Results

DXA or pQCT data were available for a total of 310 Gambian adults: 209 (M, 44%) rural and 101 (M, 51%) urban dwelling, respectively. Urban men and women were older than their rural counterparts by 3.6 and 1.6 yr, respectively (Table 1). Height, standing or sitting, did not differ in either sex by location. Urban men and women had higher values of all remaining anthropometry and DXA body composition measures, where urban participants were heavier, had greater adiposity, and a higher ratio of android to gynoid fat distribution. Mean (SD) total body percentage fat mass was greater in urban men (26.9 [7.2] vs 20 [7.2]) and women (41.0 [7.0] vs 34.6 [6.3]) compared with their rural peers. Physical function by handgrip strength was broadly similar by location for both men and women (Table 1). Rural participants of both sexes were more likely to be engaged in physical work such as farming or gardening than their urban peers (M: 85% vs 15%; F: 71% vs 32%; both *P* < .001).

**Table 1:** Descriptive characteristics of rural and urban Gambian men and women aged 55 yr and over.

	**Men**			**Women**		
	**Rural (*n* = 92)**	**Urban (*n* = 51)**	***P* value**	**Rural** **(*n* = 117)**	**Urban (*n* = 50)**	***P* value**
**Age, yr**	67.0 (8.3)	70.6 (8.4)	.02	67.3 (8.2)	68.9 (6.0)	.1
**Age categories**						
**55–64 yr** **65–74 yr** **75+ yr**	43 (46.7) 34 (37.0) 15 (16.3)	11 (21.6) 26 (51.0) 14 (27.5)	.011^a^	52 (44.4) 42 (35.9) 23 (19.7)	15 (30.0) 30 (60.0) 5 (22.0)	.014^a^
**Occupation, farming/gardening**	78 (84.8)	14 (15.2)	<.001^a^	82 (70.7)^(n = 116)^	14 (32.0)	<.001^a^
**Anthropometry**						
**Standing height (m)**	1.70 (0.07)	1.69 (0.08)	.6	1.58 (0.05)	1.57 (0.06)	.7
**Sitting height (m)**	0.84 (0.04)	0.84 (0.04)	.7	0.80 (0.03)	0.79 (0.03)	.1
**Weight (kg)**	61.3 (12.1)	71.4 (16.6)	<.001	56.4 (10.2)	68.0 (15.6)	<.001
**BMI (kg/m**^**2**^**)**	21.1 (3.7)	24.8 (4.8)	<.001	22.6 (3.7)	27.4 (6.3)	<.001
**Underweight (<18.5)** **Normal (18.5 < 25)** **Overweight (25 < 30)** **Obese (**$\mathbf{\ge}$**30)**	24 (26.1) 54 (58.7) 12 (13.0) 2 (2.2)	4 (7.8) 26 (51.0) 15 (29.4) 6 (11.8)	.00 1^a^	13 (11.1) 76 (65.0) 22 (18.8) 6 (5.1)	2 (4.0) 20 (40.0) 13 (26.0) 15 (30.0)	<.001^a^
**Waist circumference (cm)**	79.2 (11.6)	90.6 (11.5)	<.001	74.7 (9.8)	84.8 (14.1)	<.001
**Hip circumference (cm)**	89.5 (7.6)	96.7 (11.1)	<.001	89.9 (8.0)	101 (13)	<.001
**Body composition**	*n* = 92	*n* = 50		*n* = 116	*n* = 48	
**ALM (kg/m**^**2**^**)**	21.2 (4.0)	23.2 (4.2)	.007	15.1 (2.5)	17.4 (3.5)	<.001
**ALMI (kg/m**^**2**^**)**	7.30 (1.15)	8.04 (1.13)	<.001	6.04 (0.85)	7.01 (1.34)	<.001
**Total fat mass (kg)**	12.8 (6.7)	20.2 (10.0)	<.001	20.0 (6.6)	28.8 (10.4)	<.001
**FMI (kg/m**^**2**^**)**	4.42 (2.24)	6.96 (3.21)	<.001	8.00 (2.58)	11.6 (4.3)	<.001
**Android: Gynoid fat ratio**	0.41 (0.17)	0.59 (0.11)	<.001	0.34 (0.13)	0.48 (0.13)	<.001
**Functional measures**	*n* = 92	*n* = 51		*n* = 117	*n* = 50	
**HGS**	27.6 (8.2)	25.8 (7.5)	.2	18.6 (5.3)	17.7 (4.4)	.3

Data are presented as mean (SD) or *n* (%); *P* values derived from unpaired *t* test unless otherwise indicated.

^a^Chi-square test.

Abbreviations: ALM, appendicular lean mass; ALMI, appendicular lean mass index; FMI, fat mass index; HGS, handgrip strength.

### Prevalence of osteoporosis and sarcopenia

**Figure 1:**
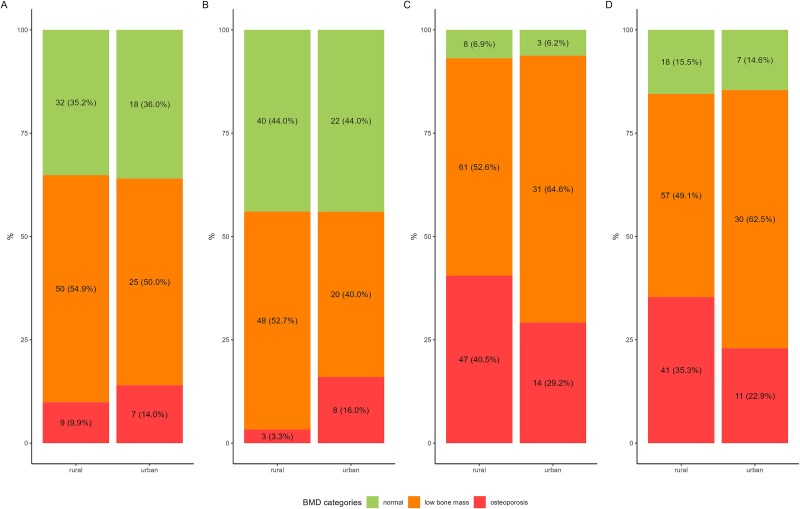
The prevalence of osteoporosis and low bone mass at the FN and TH in community-dwelling older Gambian adults by rural or urban residence: (A) male FN, (B) male TH, (C) female FN, (D) female TH. Osteoporosis was defined as a T-score <−2.5, low bone mass was defined as a T-score <1 and −2.5; all T-scores were calculated as per the International Society of Clinical Densitometry (ISCD) guidelines, using NHANES-III data for T-score calculations.

**Figure 2:**
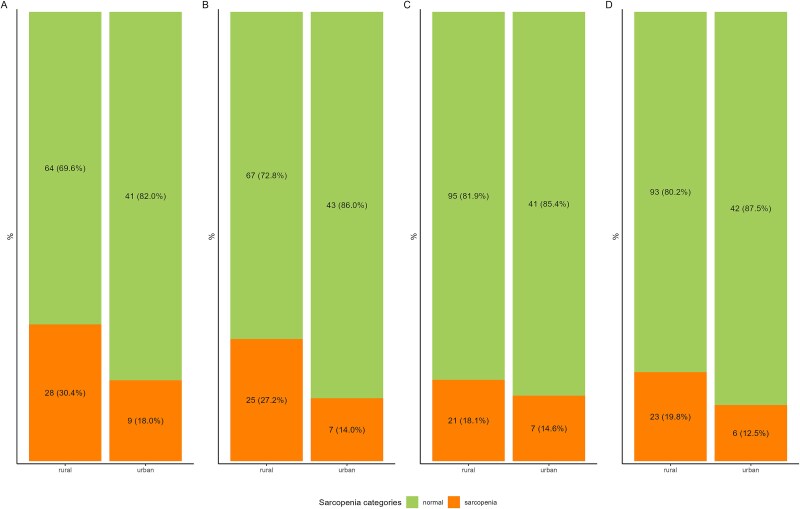
The prevalence of sarcopenia, based on the revised European Working Group on Sarcopenia in Older People (EWGSOP2) definition, in community-dwelling older Gambians. Prevalence was determined using sex-specific appendicular lean mass (ALM) or appendicular lean mass index (ALMI) cutoffs in combination with handgrip strength (HGS). (A) Men, ALM and HGS; (B) men, ALMI and HGS; (C) women, ALM and HGS; (D) women, ALMI and HGS.

The proportions of men and women with T-scores that reflect normal bone mass, low bone mass, and osteoporosis at the FN and TH by rural/urban residence are presented in Figure 1. The combined prevalence of osteoporosis at either the FN or TH was 9.9% (*n* = 9) and 20% (*n* = 10) for rural and urban men, respectively. Rural women had a greater prevalence of osteoporosis at either proximal femur site compared with urban women: 44.4% (*n* = 52) and 31.3% (*n* = 15), respectively. Low bone mass (ie, T-score between −1 and −2.5) was highly prevalent in both sexes and in both rural and urban settings (Figure 1). Lumbar spine data, from a subsample, are presented in the **Supplementary materials** and in women followed the pattern seen at the FN and TH (ie, more prevalent in rural regions). Rural men also had more osteoporosis and low bone mass at the LS, which contrasts with what was seen at the FN and TH (**Figure S1**). Sarcopenia prevalence based on EWGSOP2 definitions of ALM and grip strength was higher in rural men and women (30.4% and 18.0%, respectively) compared with their urban peers (men, 18.1%, and women, 14.6%, respectively). Using ALM normalized for height (ie, ALMI) reduced prevalence, but a similar pattern was present by sex and location. Figure 2 summarizes differences by sex and by location in ALM- and ALMI-defined sarcopenia.

### Unadjusted differences in bone density, size, and strength by location

Urban-dwelling men and women had greater DXA BA and BMC at both femoral sites (Table 2). Absolute aBMD values were greater in rural men and urban women for both proximal femur sites, although sufficient evidence was not present to reject the null hypothesis (Table 2). In contrast, LS aBMD was lower in all rural participants compared with their urban peers (Table 2).

**Table 2:** DXA and pQCT bone measures for rural and urban Gambian men and women aged 55 yr and over.

	**Men**			**Women**		
	**Rural**	**Urban**	***P* value**	**Rural**	**Urban**	***P* value**
**DXA TH**	*n* = 91	*n* = 50		*n* = 116	*n* = 48	
**T-score**	−1.08 (0.93)	−1.24 (1.02)	.4	−2.03 (1.01)	−1.90 (0.97)	.4
**aBMD (g/cm^2^)**	0.945 (0.134)	0.916 (0.143)	.2	0.752 (0.126)	0.769 (0.122)	.4
**BMC (g)**	32.6 (5.3)	33.0 (5.6)	.6	22.2 (4.1)	23.8 (4.6)	.04
**BA (cm^2^)**	34.4 (2.5)	36.0 (2.5)	<.001	29.6 (2.7)	31.1 (2.2)	<.001
**DXA FN**	*n* = 91	*n* = 50		*n* = 117	*n* = 48	
**T-score**	−1.29 (0.96)	−1.36 (0.98)	.7	−2.18 (0.78)	−2.11 (0.75)	.6
**aBMD (g/cm^2^)**	0.859 (0.133)	0.848 (0.136)	.7	0.735 (0.109)	0.745 (0.104)	.6
**BMC (g)**	4.57 (0.72)	3.83 (0.77)	<.001	3.48 (0.57)	2.95 (0.58)	<.001
**BA (cm2)**	5.34 (0.42)	5.68 (0.34)	<.001	4.81 (0.41)	5.25 (0.32)	<.001
**DXA LS**	*n* = 60	*n* = 50		*n* = 97	*n* = 48	
**T-score**	−1.53 (1.43)	−0.69 (1.73)	.008	−2.84 (1.31)	−1.72 (1.51)	<.001
**aBMD (g/cm^2^)**	1.04 (0.17)	1.14 (0.21)	.008	0.839 (0.157)	0.973 (0.181)	<.001
**Tibia 4%**	*n* = 89	*n* = 51		*n* = 116	*n* = 50	
**Total vBMD (mg/cm**^**3**^**)**	269 (42)	273 (47)	.6	215 (39)	233 (49)	.03
**Trabecular vBMD (mg/cm**^**3**^**)**	179 (44)	191 (43)	.1	138 (36)	158 (43)	.005
**Total CSA (mm**^**2**^**)**	1120 (150)	1140 (140)	.4	961 (126)	987 (111)	.2
**BSIc (g**^**2**^**/cm**^**4**^**)**	0.820 (0.250)	0.861 (0.261)	.4	0.449 (0.151)	0.549 (0.204)	.003
**Tibia 38%**	*n* = 89	*n* = 48		*n* = 114	*n* = 47	
**Cortical vBMD (mg/cm**^**3**^**)**	1230 (30)	1220 (30)	.02	1170 (50)	1180 (40)	.06
**Cortical BMC (mg/mm)**	366 (46)	379 (61)	.2	231 (47)	245 (58)	.2
**Cortical thickness (mm)**	4.92 (0.54)	4.78 (0.75)	.2	3.51 (0.67)	3.53 (0.76)	.9
**Total CSA (mm**^**2**^**)**	445 (50)	485 (64)	<.001	356 (43)	378 (51)	.01
**SSI (mm**^**3**^**)**	2010 (320)	2200 (390)	.005	1270 (250)	1380 (310)	0.03
**Radius 4%**	*n* = 76	*n* = 46		*n* = 106	*n* = 45	
**Total vBMD (mg/cm**^**3**^**)**	298 (58)	295 (55)	.8	228 (41)	236 (41)	.3
**Trabecular vBMD (mg/cm**^**3**^**)**	162 (40)	173 (42)	.2	110 (31)	117 (27)	.1
**Total CSA (mm**^**2**^**)**	414 (63)	469 (56)	<.001	360 (51)	406 (43)	<.001
**BSIc (g**^**2**^**/cm**^**4**^**)**	0.371 (0.126)	0.412 (0.132)	.1	0.189 (0.063)	0.228 (0.067)	.001
**Radius 33%**	*n* = 77	*n* = 40		*n* = 95	*n* = 30	
**Cortical vBMD (mg/cm**^**3**^**)**	1240 (30)	1240 (30)	.9	1180 (40)	1200 (40)	.02
**Cortical BMC (mg/mm)**	113 (17)	114 (17)	.7	69.7 (15.2)	73.5 (14.4)	.2
**Cortical thickness (mm)**	2.71 (0.36)	2.69 (0.38)	.8	1.87 (0.36)	1.93 (0.31)	.4
**Total CSA (mm**^**2**^**)**	135 (19)	140 (19)	.1	107 (13)	117 (13)	<.001
**SSI (mm**^**3**^**)**	330 (67)	363 (68)	.01	204 (41)	252 (44)	<.001

Data are presented as mean (SD). *P* values derived from unpaired *t* test.

Abbreviations: aBMD, areal BMD; BA, bone area; BSIc, bone strength index of compression; CSA, cross-sectional area; pQCT, peripheral QCT; SSI, stress-strain index; vBMD, volumetric BMD.

In men, there was no evidence of rural–urban differences in tibia vBMD, size, or strength (Table 2). Urban women had greater vBMD, CSA, and BSIc than rural women at the distal tibia (Table 2). At the proximal tibia, urban men had lower cortical vBMD, total CSA, and stress-strain index (SSI) compared with their rural counterparts (Table 2). This pattern was broadly consistent in women, except for cortical vBMD (Table 2). At the distal radius, urban men had greater CSA and urban women had greater CSA and BSIc compared with rural participants (Table 2). At the proximal radius, urban participants had greater SSI than their rural peers, while urban women also had greater cortical vBMD and CSA (Table 2).

### Adjusted between-group differences in bone density, size, and strength

#### DXA

**Figure 3:**
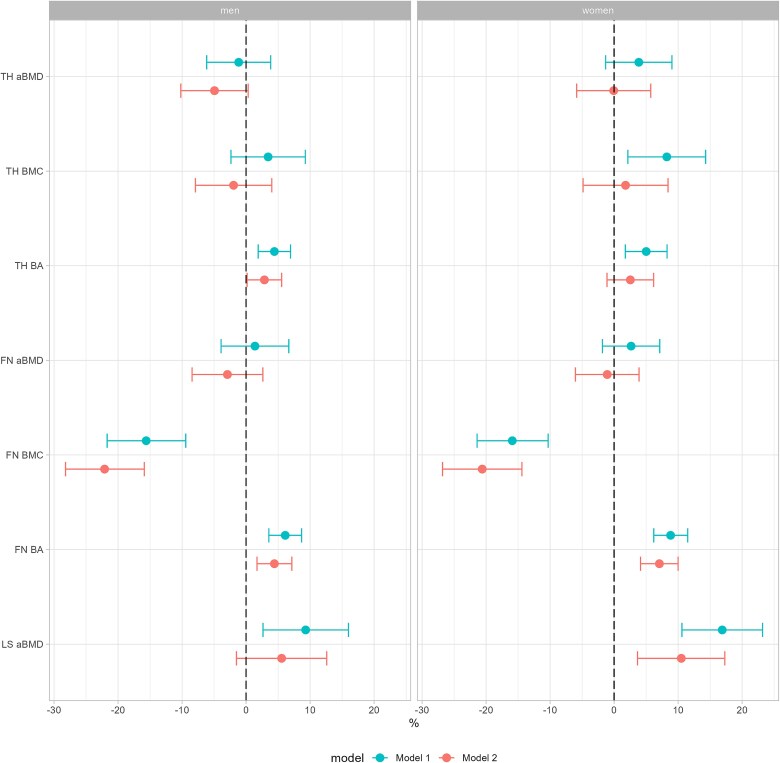
Rural–urban differences in DXA outcomes at the FN, TH, and LS in older Gambian adults by sex. Model 1 adjusted for age; model 2 adjusted for age and fat mass index (FMI). Beta-coefficients are interpretable as the symmetric percentage difference in urban DXA values from those of their rural peers.

In men, after adjusting for age, rural–urban differences in DXA bone measures were broadly consistent with the unadjusted data: urban men had greater TH BA and FN BA (4.4% [1.9%–7.0%] and 6.1% [3.6%–8.7%], respectively), but −15.6% (−21.7% to −9.4%) lower FN BMC (Figure 3, Table S2). Lumbar spine aBMD was 9.3% (2.7%-16.0%) greater in urban men. After further adjustment for FMI, the difference in TH and FN aBMD strengthened, with urban men having lower aBMD than rural men (Figure 3, Table S2). The greater LS aBMD of urban men was attenuated (Figure 3, Table S2).

In women, age adjustment had minimal impact on the rural–urban DXA differences described above: urban women had greater TH BMC and BA of 8.3% (2.2%–14.4%) and 5.1% (1.9%–8.4%), respectively; lower FN BMC and higher BA of −15.8% (−21.3% to −10.2%) and 8.9% (6.3%–11.5%), respectively (Figure 3, Table S2). Lumbar spine aBMD was 16.9% (10.6%-23.2%) greater in urban women. Further adjustment for FMI attenuated all rural–urban differences in all TH outcomes and FN aBMD, but differences in LS aBMD, FN BMC, and BA were robust to adjustment (Figure 3, Table S2). Observed patterns at FN and TH outcomes remained consistent when repeated in the subset with LS aBMD data (Figure S2).

### pQCT

**Figure 4:**
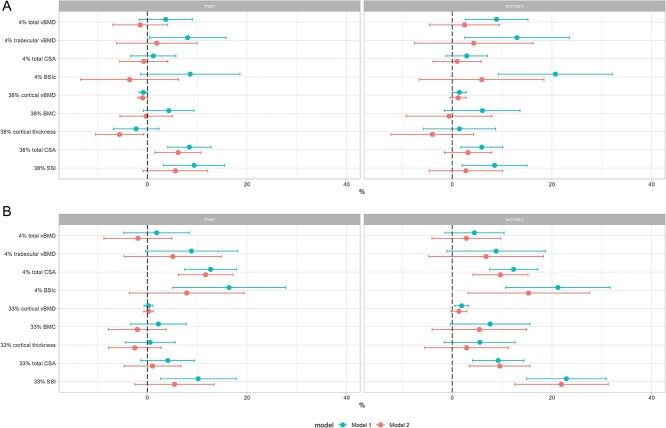
Rural–urban differences in peripheral QCT (pQCT) outcomes at the (A) tibia and (B) radius in older Gambian adults by sex. Model 1 adjusted for age; model 2 adjusted for age and fat mass index (FMI). Beta-coefficients are interpretable as the symmetric percentage difference in urban pQCT values from those of their rural peers. Abbreviations: BSIc, bone strength index of compression; CSA, cross-sectional area; SSI, stress-strain index; vBMD, volumetric BMD.

After adjusting for age, urban men had 8.1% (0.4%-15.8%) greater distal tibial trabecular and total vBMD than rural men. Further adjustment for FMI attenuated these associations (Figure 4A, Table S3). Urban men had greater, age-adjusted, proximal tibia CSA and SSI compared with rural men; cortical vBMD and thickness were lower but this failed to reach statistical significance. After adjustment for FMI, tibia CSA was greater, and cortical vBMD and cortical thickness were lower, in urban men (Figure 4A, Table S3).

Urban women had greater age-adjusted total vBMD, trabecular vBMD, and BSIc of 8.9% (2.6%-15.1%), 13.1% (2.6%-23.5%), and 20.7% (9.3%-32.1%), respectively, compared with their rural counterparts (Figure 4A, Table S2). Age-adjusted proximal tibia cortical vBMD, total CSA, and SSI were higher in urban women (Figure 4A, Table S3). All female rural–urban differences at the tibia were attenuated once FMI was accounted for (Figure 4A, Table S3).

Rural–urban differences were evident in men at the distal radius, where, in urban men, total CSA and BSIc were greater by 12.7% (7.5%-17.9%) and 16.4% (5.2%-27.8%), respectively (Figure 4B, Table S3). Total CSA, but not SSI, was robust after FMI adjustment (Figure 4B, Table S3). Urban men had 10.2% (2.7%-17.8%) greater age-adjusted proximal radius SSI, although this was attenuated following further adjustment for FMI.

Urban women also had greater age-adjusted distal radius CSA and BSIc of 12.4% (7.6%-17.1%) and 21.3% (10.8%-31.7%), respectively. Age-adjusted radius cortical vBMD, total CSA, and SSI were 1.9% (0.5%-3.2%), 9.3% (4.2%-14.5%), and 23.1% (12.5%-31.0%) higher in urban women, respectively. Except for cortical vBMD, all radius differences were robust to FMI adjustment (Figure 4B, Table S3). The pattern of findings was broadly consistent when the above pQCT models were run on the smaller complete cases subset (Figure S3).

## Discussion

In this, the first rural–urban comparison of osteoporosis and sarcopenia prevalence in Africa, we found that, while the burden of osteoporosis and sarcopenia varied by both location and sex, low bone mass (“osteopenia”) was highly prevalent. Older rural Gambian men were more likely to have sarcopenia than osteoporosis, with urban men having greater osteoporosis prevalence at all sites. In women, musculoskeletal health deficits were more consistent, as those in rural areas had a higher prevalence of osteoporosis (any site) and sarcopenia. In all subgroups, osteoporosis was most common at the LS. In addition to DXA, pQCT was used to describe phenotypic differences in bone density, mass, and geometry adjusted for age and FMI. Overall, we found a broadly consistent pattern of rural–urban bone differences within sexes. Urban men had larger bones and lower mineral content, which possibly explains the relative lack of difference in FN and TH aBMD (which is derived from BA and BMC) when age and FMI were adjusted for. There was also a tendency for greater LS aBMD in urban men, which was attenuated by FMI adjustment. Tibial pQCT findings were similar to the femoral DXA, CSA was greater in urban men, and despite thinner cortices and lower cortical vBMD (likely due to thinner cortices and the partial volume effect), estimated strength was greater. Rural–urban DXA differences in women were largely similar to those in men, although a stronger relationship was seen for LS aBMD, which was 10% higher in urban women and robust to adjustment for FMI and age. All radial pQCT measures of CSA and strength were greater in urban women.

### Contextualization of our findings in relation to future fracture risk

As documented previously in The Gambia, greater adiposity is becoming a key trait of urban residence,^17^^,^^18^^,^^34^ particularly in women. While the influence of greater body size on bone is relatively well established, evidence of the impact of adiposity on fragility fracture continues to accumulate, with obesity now considered a risk factor in some populations.^35^ The current findings provide insight into the potential impact of environment and place of residence on musculoskeletal health and phenotype, which may influence falls and fracture. Notably, participants shared a common ethnic background and urban participants spent their formative years in the rural setting, when the majority of their musculoskeletal development (ie, attainment of peak bone and muscle mass) will have occurred. This is reflected by similar standing and sitting height, suggesting similar exposures during growth influencing pubertal timing.^36^ Rural–urban differences in adult bone therefore are likely driven by a constellation of factors not limited to urban lifestyle and diet, which predispose to greater weight gain and adiposity across midlife. Our data suggest that rural–urban differences in aBMD at the hip (FN and TH) are largely explained by age and FMI; however, interestingly, differences in BA and mineralization were not. Furthermore, it was notable that rural participants had markedly lower LS aBMD, and these differences were robust to adjustment for differences in adiposity. Given recent findings of a prevalence of vertebral fracture similar to that across the globe, and that BMD was an important risk factor for prevalent and incident fractures in the rural participants, this raises important concerns for management of future fracture risk in rural communities. Further work needs to establish modifiable risk factors. It should be noted that the accuracy of DXA aBMD estimates can be influenced by the amount of fat tissue, which could result in some residual confounding of these relationships due to the particularly high rates of obesity in urban women^37^; adjustment for FMI was the most robust way to adjust in this population. Using pQCT measurements of the tibia showed that the distribution of bone (larger bones, thinner cortices) was altered, leading to greater strength. The limitations of aBMD in populations of differing race and ethnicity are well documented and how this contributes to fracture risk needs to be determined, as is the need for robust epidemiological data on fracture prevalence and incidence to determine the relationships between BMD and fracture risk.^38^

### Sex-specific patterns of osteoporosis and sarcopenia

The rural–urban pattern of osteoporosis and sarcopenia prevalence differed within sexes but the investigation was constrained by fewer explanatory variables, such as physical activity and diet, in the urban population. A likely explanation for the differing prevalence of osteoporosis in men and women relates to age and body composition. Urban men were approximately 3 years older than rural men, with 27.5% vs 16.3% aged over 75 yr. As osteoporosis risk increases with age,^5^ this likely accounts for the pattern we observed in T-score–defined osteoporosis, compared with the smaller differences in aBMD in our age-adjusted models. In contrast, among women, where age distribution was similar, the pattern we saw is consistent with the rural–urban differences in body composition. Greater body mass enhances skeletal loading, which, in turn, can favor higher BMD. It is telling that, in our models, female rural–urban differences in aBMD (FN and TH) and tibial pQCT outcomes were largely attenuated when body composition was accounted for. Equally, FMI was positively associated with those outcomes in women, whereas the relationships were less consistent in men. From a muscle-health perspective, a greater sarcopenia prevalence among rural participants of both sexes may seem counterintuitive. However, national survey data suggest that the consumption of animal-protein food groups (ie, beef and lamb, poultry, and eggs) is greater in urban compared with rural areas.^21^ This may suggest that, while rural participants may be less sedentary than urban peers, they have poorer access to certain protein-rich food groups, which may partially explain what we observed in lean mass. In the rural GamBAS cohort, previous efforts to model nutrition (via dietary patterns) have been ineffective as the rural diet is very homogenous. Ongoing studies with more balanced rural–urban sampling will help determine the generalizability of these preliminary findings.^25^

### Contextualization of our findings to international data

These are the first data to document rural and urban difference in bone density, mass, geometry, and strength in African older adults using gold-standard X-ray methodology. Elsewhere, small studies, comparing bone health in rural–urban settings in Namibia and Nigeria, have been conducted using quantitative ultrasound (QUS), which assesses parameters related to bone density and quality but cannot be used to diagnose osteoporosis.^39^ In Namibia, rural and urban Ovahimba people (*n* = 98; F: 70%) were compared, although participants were younger than in the present study^40^ and sex-specific rural–urban QUS differences are impossible to discern as men and women were pooled. It was reported that urban dwellers had lower speed of sound (SOS) and stiffness index (SI), related to bone density, following adjustment for sex, age, height, and weight.^40^ Similar work from Nigeria compared semi-nomadic Fulani rural-dwelling men (*n* = 51) with previously collected data from urban Nigerians.^41^^,^^42^ Matsuzaki and colleagues^28^ included 4 studies from LMICs in their 2015 meta-analysis, concluding that 3 out of 4 studies from LMICs provided evidence that BMD in urban areas is higher than in rural areas.

To date, there have been relatively few studies directly assessing differences in sarcopenia prevalence between rural and urban populations.^43–46^ While a recent systematic review and meta-analysis has been published on this topic, few of the included studies included both rural and urban samples.^47^ Comparisons of sarcopenia prevalence in the current study with other African studies, or indeed studies from other LMICs, is hampered by the lack of clear population-specific diagnostic criteria for sarcopenia in Africans. The reported prevalence of sarcopenia in the current study is similar to the mean weighted prevalence (25.7%) reported in a recent meta-analysis in African older people. However, of the 6 studies that met the inclusion criteria, 2 publications were from baseline data from the GamBAS cohort. The studies included were heterogeneous, using differing definitions of sarcopenia, which is a major limitation in strengthening the evidence base. Data from Burkina Faso suggest that physical performance values worse than the cutoff values in the current European sarcopenia guidelines are common,^48^ whereas, in contrast, a previous study of rural South African women, using EWGSOP2, no participant had confirmed sarcopenia; probable sarcopenia affected more men than women (11.6% vs 4.4%).^49^ Notwithstanding, in our current comparison, which follows the EWGSOP2 definitions, there were clear rural–urban differences in men and in women, with a higher prevalence in rural-dwelling older men. Interesting, this is in keeping with findings from western India,^43^ where they applied the Asian Working Group on Sarcopenia definition. Understanding the underlying determinants of sarcopenia in these populations is of utmost importance given the rapidly rising aging population in The Gambia and other LMICs. As such, to meet this urgent need for reliable diagnostic criteria in resource-limited settings, there is a necessity to move away from lean mass measures that rely on expensive and scarce technology (eg, DXA) toward implementable solutions based on affordable function-based criteria.

### Strengths and limitations

This is the first rural–urban comparison of osteoporosis, sarcopenia, DXA-assessed bone density, and pQCT-measured bone architecture in Africa. By study design, participants in this study were drawn from the same ethnic background and shared largely similar early-life exposures in rural subsistence-farming communities. In a multiethnic country such as The Gambia, this minimizes the impact of potential genetic differences being the primary driver of rural–urban differences in musculoskeletal health. Equally, all imaging technology was cross-calibrated using in vivo data collected close in time to the present study.^31^ However, this study has limitations. Our overall findings may not be generalizable to the whole population; the focus here was on 2 regions and predominantly the Mandinka ethnic group (Sukuta is one of the most densely populated urban conurbations of The Gambia). Urban recruitment occurred at a different time to initial rural recruitment, and recruitment methods differed due to a lack of an urban Demographic Surveillance System, which meant that convenience sampling was used. Urban recruitment was weighted toward older ages (60-80 yr), making the mean age slightly older than in the rural region. And, while age was adjusted for in all models, we cannot fully rule out potential residual confounding. Urban women lacked reproductive history data (ie, parity, breastfeeding, and detailed menopausal staging), although our minimum age of 55 yr ensured that women were postmenopausal. We also lacked data on habitual diet (eg, vitamin D and calcium intake), 25(OH)D status, and other laboratory measures, previously shown to be risk factors for bone loss and fracture. Our use of the EWGSOP2 definition of sarcopenia was pragmatic given the lack of population-specific definitions available for African populations. Due to the nature of pQCT scanning and the demographics of our participant cohort (ie, older adults), there was a much greater loss of data at the radius due to movement artifact than at any of the other skeletal sites measured. Also, due to computer failure, a proportion of DXA LS scans were not available for the present analysis.

### Conclusions

Low bone mass is highly prevalent in older Gambian adults of both sexes, regardless of rural or urban residence, although the within-sex burden of osteoporosis differed by rural–urban residence. Urban men and rural women were more likely to meet the international criteria for osteoporosis diagnosis. Sarcopenia prevalence was greatest in rural men. Most rural–urban differences in DXA and pQCT measures of bone density, mass, geometry, and strength were not independent of FMI, suggesting that nutrition, both undernutrition and overnutrition, and loading play important roles in this setting. The notable exception to this was bone size and strength at the radius, which was greater in urban women, independent of adiposity (FMI). Given the paucity of epidemiological musculoskeletal data from African countries coupled with ongoing nutrition transition and urbanization, larger population studies are urgently required to accurately describe the prevalence of osteoporosis and sarcopenia. This would enable the better prediction of adverse musculoskeletal outcomes using population-specific criteria.

## Supplementary Material

ASBMR-25020145_Supplementary_materials_2025_07_04_zjaf130

## Data Availability

Data are available upon reasonable request from the senior author.
