# Supplementary material for: Rural–urban differences in osteoporosis and sarcopenia prevalence among Gambian older adults: a pilot study
Source: J Bone Miner Res. 2025 Sep 22;41(8):804–14. doi: 10.1093/jbmr/zjaf130 (PMC13421071; doi:10.1093/jbmr/zjaf130)

**Supplementary materials**

**Table S1.** Missingness of data due to poor scan quality, machine breakdown, or scan artifacts for participants included in the analysis with at least one usable DXA or pQCT scan (n = 310).

|  | Men | | Women | |
| --- | --- | --- | --- | --- |
|  | Rural (n=92) | Urban (n=51) | Rural (n=117) | Urban (n=50) |
| DXA | | | | |
| Total body | 0 | 1 | 1 | 2 |
| Total hip | 1 | 1 | 1 | 2 |
| Femoral neck | 1 | 1 | 1 | 2 |
| Lumbar spine | 31 | 1 | 20 | 2 |
| pQCT | | | | |
| Radius 4% | 16 | 5 | 11 | 5 |
| Radius 33% | 15 | 11 | 22 | 20 |
| Tibia 4% | 3 | 0 | 1 | 0 |
| Tibia 38% | 3 | 3 | 3 | 3 |
| 4 rural participants, 3 men and 1 woman, had no available scan data and were not included in the analyses. | | | | |

**Table S2.** Rural-urban differences in dual-energy x-ray absorptiometry (DXA) outcomes at the femoral neck (FN) and total hip (TH) of older Gambian adults by sex. Model 1 adjusted for age, Model 2 adjusted for age and fat mass index (FMI). Beta-coefficients interpretable as the symmetric percentage difference in urban DXA values from those of their rural peers.

|  | DXA | Model 1 | | Model 2 | |
| --- | --- | --- | --- | --- | --- |
|  | variable | Sym% [95%CI] | p-value | Sym% [95%CI] | p-value |
| Men | TH aBMD | -1.2 [-6.2; 3.9] | 0.650 | -4.9 [-10.2; 0.3] | 0.066 |
|  | TH BMC | 3.5 [-2.4; 9.3] | 0.243 | -2.0 [-7.9; 4.0] | 0.520 |
|  | TH BA | 4.4 [1.9; 7.0] | 0.001 | 2.9 [0.2; 5.6] | 0.037 |
|  | FN aBMD | 1.4 [-3.9; 6.7] | 0.604 | -2.9 [-8.4; 2.6] | 0.300 |
|  | FN BMC | -15.6 [-21.7; -9.4] | <0.001 | -22.1 [-28.2; -15.9] | 0.000 |
|  | FN BA | 6.1 [3.6; 8.7] | <0.001 | 4.4 [1.7; 7.2] | 0.002 |
|  | LS aBMD | 9.3 [2.7; 16.0] | 0.007 | 5.6 [-1.5; 12.6] | 0.121 |
| Women | TH aBMD | 3.8 [-1.3; 9.0] | 0.143 | -0.1 [-5.8; 5.7] | 0.984 |
|  | TH BMC | 8.3 [2.2; 14.4] | 0.008 | 1.8 [-4.9; 8.4] | 0.595 |
|  | TH BA | 5.1 [1.9; 8.4] | 0.002 | 2.5 [-1.1; 6.2] | 0.170 |
|  | FN aBMD | 2.7 [-1.8; 7.1] | 0.235 | -1.1 [-6.0; 3.9] | 0.670 |
|  | FN BMC | -15.8 [-21.3; -10.2] | <0.001 | -20.6 [-26.8; -14.4] | <0.001 |
|  | FN BA | 8.9 [6.3; 11.5] | <0.001 | 7.1[4.1; 10.0] | <0.001 |
|  | LS aBMD | 16.9 [10.6; 23.2] | <0.001 | 10.5 [3.7; 17.3] | 0.003 |
| Model 1= age adjusted, Model 2= age and FMI adjusted. Sym% = symmetric percentage, 95%CI = 95% confidence interval, vBMD, aBMD =areal bone mineral density, BMC = bone mineral content, BA = bone area. | | | | | |

**Table S3.** Rural-urban differences in peripheral Quantitative Computed Tomography (pQCT) outcomes at the tibia and radius in older Gambian adults by sex. Model 1 adjusted for age, Model 2 adjusted for age and fat mass index (FMI). Beta-coefficients interpretable as the symmetric percentage difference in urban pQCT values from those of their rural peers.

|  |  | **TIBIA** | | | | **RADIUS** | | | |
| --- | --- | --- | --- | --- | --- | --- | --- | --- | --- |
|  |  | Model 1 | | Model 2 | | Model 1 | | Model 2 | |
|  | variable | Sym% [95%CI] | p-value | Sym% [95%CI] | p-value | Sym% [95%CI] | p-value | Sym% [95%CI] | p-value |
| Men | Total vBMD | 3.7 [-1.6;9.0] | 0.170 | 1.9 [-6.2; 10.0] | 0.643 | 1.9 [-4.7;8.5] | 0.574 | -1.9 [-8.7; 4.9] | 0.584 |
|  | Trabecular vBMD | 8.1 [0.4;15.8] | 0.039 | -1.4 [-6.9; 4.1] | 0.610 | 8.8 [-0.4;18.1] | 0.060 | 5.1 [-4.7; 14.9] | 0.303 |
|  | Total CSA | 1.2 [-3.3;5.7] | 0.601 | -0.7 [-5.5; 4.1] | 0.767 | 12.7 [7.5;17.9] | <0.001 | 11.7 [6.2; 17.2] | <0.001 |
|  | BSIc | 8.6 [-1.4;18.6] | 0.09 | -3.6 [-13.4; 6.3] | 0.475 | 16.4 [5.2;27.8] | 0.005 | 7.9 [-3.6; 19.4] | 0.176 |
|  | Cortical vBMD | -0.8 [-1.7;0.1] | 0.065 | -1.0 [-1.9; -0.0] | 0.046 | 0.2 [-0.7;1.1] | 0.621 | 0.3 [-0.7; 1.2] | 0.600 |
|  | Cortical BMC | 4.3 [-0.8;9.4] | 0.097 | -0.2 [-5.5; 5.0] | 0.930 | 2.2 [-3.4;7.8] | 0.432 | -2.0 [-7.8; 3.8] | 0.495 |
|  | Cortical thickness | -2.3 [-6.8;2.3] | 0.333 | -5.6 [-10.4; -0.7] | 0.025 | 0.6 [-4.4;5.6] | 0.823 | -2.5 [-7.8; 2.7] | 0.347 |
|  | Total CSA | 8.4 [4.1;12.8] | <0.001 | 6.2 [1.5; 10.8] | 0.009 | 4.1 [-1.3;9.5] | 0.136 | 1.0 [-4.7; 6.7] | 0.719 |
|  | SSI | 9.4 [3.3;15.5] | 0.003 | 5.6 [-0.9; 12.1] | 0.089 | 10.2 [2.7;17.8] | 0.009 | 5.5 [-2.5; 13.4] | 0.178 |
| Women | Total vBMD | 8.9 [2.6;15.1] | 0.006 | 2.5 [-4.6; 9.5] | 0.491 | 4.5 [-1.4;10.3] | 0.138 | 2.8 [-4.1; 9.7] | 0.421 |
|  | Trabecular vBMD | 13.1 [2.6;23.5] | 0.014 | 4.3 [-7.6; 16.2] | 0.476 | 8.9 [-1.0;18.7] | 0.076 | 6.8 [-4.7; 18.3] | 0.244 |
|  | Total CSA | 3.0 [-1.2;7.1] | 0.162 | 1.0 [-3.9; 5.8] | 0.692 | 12.4 [7.6;17.1] | <0.001 | 9.7 [4.1; 15.2] | 0.001 |
|  | BSIc | 20.7 [9.3;32.1] | <0.001 | 5.9 [-6.6; 18.4] | 0.354 | 21.3 [10.8;31.7] | <0.001 | 15.3 [3.2; 27.5] | 0.014 |
|  | Cortical vBMD | 1.4 [0.1;2.8] | 0.037 | 1.2 [-0.4; 2.8] | 0.140 | 1.9 [0.5;3.2] | 0.006 | 1.3 [-0.3; 2.9] | 0.105 |
|  | Cortical BMC | 6.1 [-1.4;13.7] | 0.112 | -0.7 [-9.3; 7.9] | 0.881 | 7.8 [-0.2;15.7] | 0.057 | 5.4 [-4.1; 14.9] | 0.259 |
|  | Cortical thickness | 1.5 [-5.7;8.7] | 0.683 | -4.0 [-12.3; 4.3] | 0.342 | 5.7 [-1.4;12.7] | 0.116 | 2.9 [-5.6; 11.3] | 0.500 |
|  | Total CSA | 6.0 [1.8;10.1] | 0.005 | 3.2 [-1.6; 7.9] | 0.192 | 9.3 [4.2;14.5] | 0.001 | 9.5 [3.5; 15.6] | 0.002 |
|  | SSI | 8.6 [2.1;15.1] | 0.010 | 2.7 [4.6; 10.1] | 0.465 | 23.1 [15.1;31.0] | <0.001 | 21.9 [12.5; 31.3] | <0.001 |
| Model 1= age adjusted  Model 2= age and FMI adjusted  Sym% = symmetric percentage, 95%CI = 95% confidence interval, vBMD = volumetric bone mineral density, CSA = cross-sectional area, BSIc = bone strength index of compression, SSI = stress-strain index | | | | | | | | | |

**Figure S1.** The prevalence of osteoporosis and low bone mass at the lumbar spine in community dwelling older Gambian adults by rural or urban residence: A) men, B) women. Osteoporosis defined as a T-score <2.5, low bone mass defined as a T-score <1 and >2.5, all T-scores calculated as per ISCD guidelines, using NHANES III data for T-score calculations.


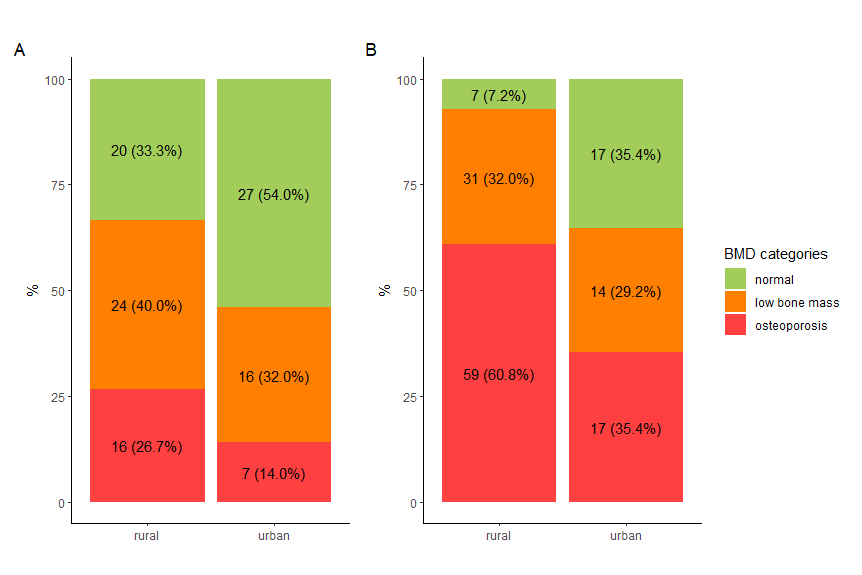


**Figure S2.** Rural-urban differences in dual-energy x-ray absorptiometry (DXA) outcomes at the femoral neck (FN), total hip (TH), and lumbar spine (LS) of older Gambian adults by sex. Model 1 adjusted for age, Model 2 adjusted for age and fat mass index (FMI). **Complete cases (n=255) sensitivity analysis performed due to missingness in LS data.** Beta-coefficients interpretable as the symmetric percentage difference in urban DXA values from those of their rural peers. aBMD = areal bone mineral density, BMC = bone mineral content, BA = bone area.

**
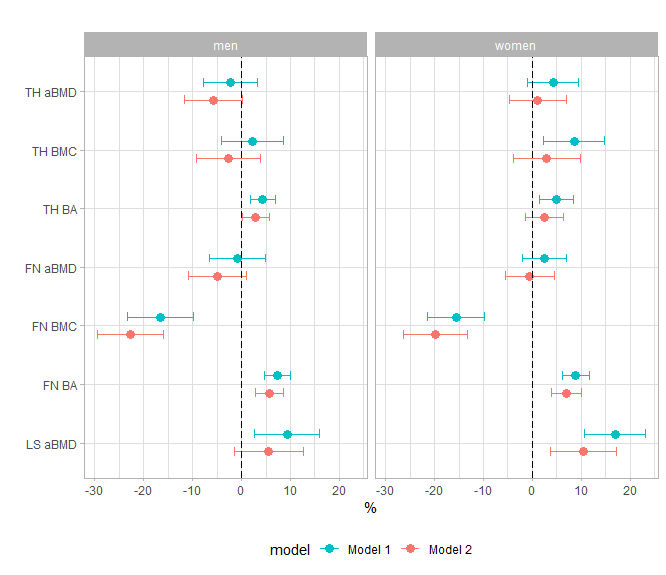
**

**Figure S3.** Rural-urban differences in peripheral Quantitative Computed Tomography (pQCT) outcomes at the A) tibia and B) radius in older Gambian adults by sex. Model 1 adjusted for age, Model 2 adjusted for age and fat mass index (FMI). **Complete cases (n=212) sensitivity analysis performed due to missingness in radial pQCT data.** Beta-coefficients interpretable as the symmetric percentage difference in urban pQCT values from those of their rural peers. vBMD = volumetric bone mineral density, CSA = cross-sectional area, BSIc = bone strength index of compression, SSI = stress-strain index.


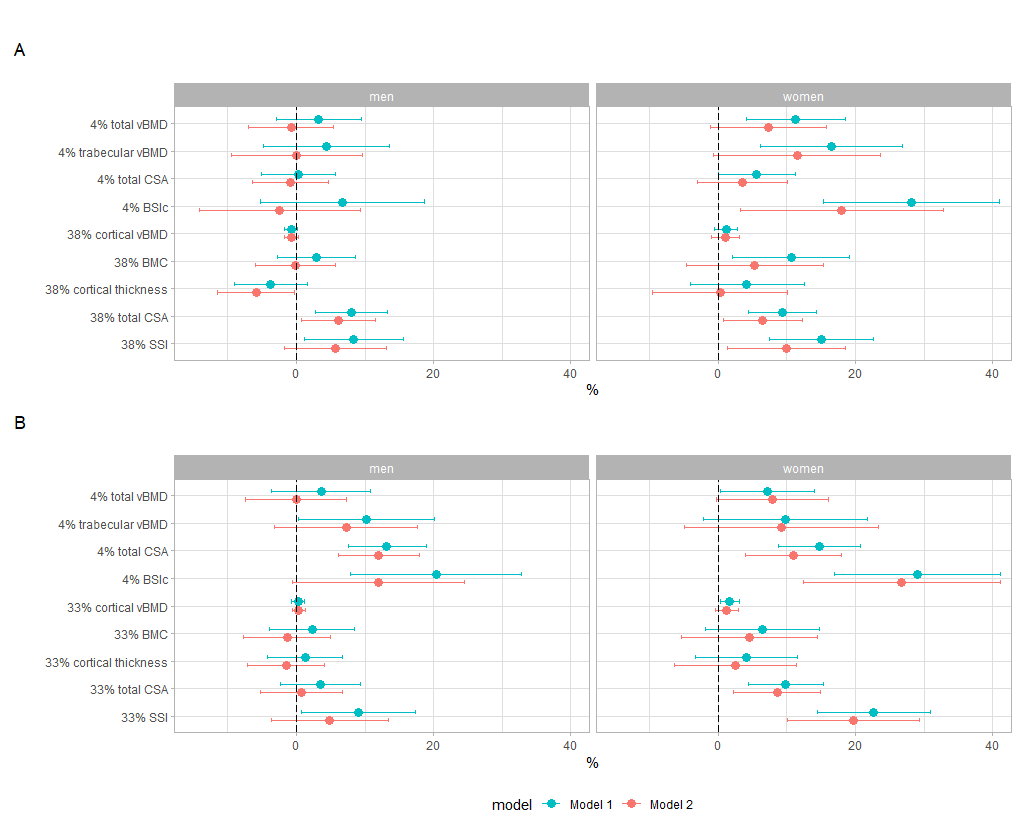

Supplement: ASBMR-25020145_Supplementary_materials_2025_07_04_zjaf130 [file asbmr-25020145_supplementary_materials_2025_07_04_zjaf130.docx]
